# Knowledge, Practice and Attitudes to the Management of Sepsis in Jamaica

**DOI:** 10.2478/jccm-2022-0024

**Published:** 2022-11-12

**Authors:** Karen Roye-Green, Rohan Willis, Sharon R. Priestley, Ivan Vickers

**Affiliations:** 1 The University of the West Indies Mona Campus, Department of Microbiology, Kingston, Jamaica; 2 The University of Texas Medical Branch at Galveston Department of Internal Medicine, TX, USA; 3 The University of the West Indies Mona Campus, Department of Sociology, Psychology and Social Work, Kingston, Jamaica

**Keywords:** knowledge, practice, attitudes, critical care survey, sepsis bundles

## Abstract

**Introduction:**

Sepsis is a life-threatening dysfunction resulting from the dysregulated host response to infection. The mortality of sepsis in Jamaica remains high amid the proven efficacy of the Surviving Sepsis Guidelines implementation in some countries.

**Aim of study:**

To evaluate the inter-relationship of healthcare workers’ attitude towards, knowledge of and practice of sepsis management in Jamaica.

**Material and methods:**

A survey was done using an anonymous self-administered validated questionnaire to healthcare workers across Jamaica. Questions on knowledge, attitude, and practice of sepsis within private and public hospitals were answered.

**Results:**

A total of 616 healthcare workers were eligible for analysis. Most respondents agree that healthcare workers need more training on sepsis (93.7%) and that formal sepsis training modules should be implemented at their hospitals or practice (93.2%). Several signs of sepsis as outlined by qSOFA were correctly identified as such by most respondents (60.6% to 76.4%), with the exception of a low PaCO2 (34.9%), which was correctly identified by a minority of respondents. While a majority (69.3%) were able to correctly define sepsis, only 8.8% of respondents knew the annual sepsis mortality rate. Postgraduate training (p<0.01) and formal sepsis training (p<0.05) were both predictive of high correct knowledge and practice scores. Specialization in Anaesthesia/ Critical Care Medicine (p<0.05) or Emergency Medicine (p<0.05) was predictive of high knowledge scores and Internal Medicine predictive of high practice scores (p<0.01).

**Conclusions:**

This study revealed that education for healthcare workers on sepsis and the implementation of SSC is needed in Jamaica.

## Introduction

Sepsis is a global health problem, causing one in five deaths around the world. In 2017, an estimated 48.9 million incident cases of sepsis were reported worldwide with 11.0 million sepsis-related deaths [1]. These estimates include data for both adult and children from high-, middle- and low-income countries in contrast to previous estimates that accounted only for reports in adults from first world countries. Sepsis is the leading cause of in-hospital deaths with an annual estimated cost of 24.3 billion dollars in the USA [2,3].

Preventive measures are key to reducing the burden of infection and these include good hand hygiene, vaccination, and proper sanitation. However, the management of infected persons necessitates prompt identification of the condition to facilitate timely intervention that will improve clinical outcomes. The presentation of sepsis may include a wide variety of signs and symptoms which can be hard to detect. The recognition of a septic patient and the start of appropriate antibiotics are critical decisions made by clinicians. These decision-making skills are often more practiced and nuanced by critical care clinicians, than other generalist physicians.

Rapid diagnosis and management of sepsis is critical for good outcome [4, 5, 6, 7]. Failure to early identify the patient with sepsis which might be difficult, can result in treatment delay [8, 9, 10]. It has been shown that even with implementation of sepsis protocols, the percentage of healthcare workers who are knowledgeable in this regard remains low [11, 12, 13]. However, with early treatment of sepsis a decrease in mortality and morbidity is possible [10,14].

Studies which have been done to highlight the variability in healthcare workers’ knowledge of sepsis have utilized surveys and analyzed results according to their varied demographics and clinical training [15]. For this study, we aimed to study the inter-relationship of healthcare workers’ attitudes towards, knowledge of and practice of sepsis management in Jamaica.

## Methods

### Ethical approval

Our study was approved by the University Hospital of the West Indies/ University of the West Indies (UHWI/ UWI) Faculty of Medical Sciences Ethics Committee (Mona) (ECP 32, 16/17) and ethics committee of Ministry of Health of Jamaica (2016/31).

### Questionnaire Development and Administration

A 25-item questionnaire was developed by the researchers and adjustments were made after a pilot study. The first 9 questions were on demographics (occupation, years post registration, post graduate specialty if applicable, gender, type of healthcare institution). All the other questions were in a multiple-choice format and assessed knowledge of, attitude towards and typical practice of sepsis management based on the 2016 International Sepsis Guidelines [16].

We performed a prospective, cross-sectional study using the self-administered questionnaire between June 15, 2018, and June14, 2019. All healthcare workers (HCWs) throughout Jamaica were eligible for participation. Participants were recruited by convenience sampling among conference attendees and registrants at the Medical Council of Jamaica. The study was explained to each participant who completed the questionnaire after giving informed consent.

### Assessment of Respondent Attitudes Toward Sepsis Management

To assess healthcare workers’ attitudes toward sepsis management, three questions (Q15-17) evaluated the extent to which respondents agree that healthcare workers can identify patients at great risk for sepsis as well as the need for more sepsis training.

### Assessment of Respondent Knowledge and Practice of Sepsis Management

The assessment of healthcare workers’ knowledge and practice of sepsis management was performed quantitatively. Each answer for factual knowledge or practice questions was allocated a value of 1 point and then separated into correct or incorrect groups and scores tabulated.

Factual knowledge questions (Q10-13) had a total of 7 correct answers and so was scored out of a maximum of 7 points. Question 10 had 3 correct answers, question 12 had 2 correct answers and question 11 and 13 each had 1 correct answer. These knowledge questions also had a total of 7 incorrect answers and had a maximum score of 7 points. Question 10 had 2 incorrect answers, question 11 had 3 incorrect answers, and questions 12 and 13 were each scored as 1 point.

Factual practice questions (Q18-24) had a total of 14 correct answers and so was scored out of a maximum of 7 points. Question 23 had 4 correct answers, questions 18 and 22 each had 3 correct answers and questions 19, 20, 21 and 24 each had 1 correct answer. These practice questions had a total of 7 incorrect answers and so had a maximum score of 7 points. All questions 18-24 each had 1 incorrect answer.

Mean values for each of the 4 calculated scores were calculated for each respondent group to facilitate comparisons and to determine demographic and clinical characteristics that predict higher or lower scores. Note that high correct scores indicate appropriate knowledge or practice for sepsis management, while high incorrect scores indicate inappropriate knowledge or practice in this regard.

### Statistical analysis

Statistical analyses were performed using the Xlstat Software, Version 16.6 (Addinsoft, New York, NY). The significance in difference of score means and comparisons of outcomes across respondent groups were calculated by Student’s t-test or ANOVA, with post-hoc multiple pairwise comparisons using Tukey test as appropriate. The lowest scoring group(s) in each characteristic variable served as the baseline reference for the Tukey pairwise comparisons. Multivariate forward stepwise linear regression analysis was used to determine characteristics that are independent predictors for high or low values of the 4 scores. A probability limit of 0.1 was used for variable entry into the linear regression model and the suitability of the resultant model was illustrated by the calculated R^2^ and adjusted R^2^ values. Results were considered statistically significant if p values were less than 0.05 (p<0.05).

## Results

### Respondent profile

A total of six-hundred and sixteen (n=616) clinical healthcare workers participated in the study. The overall response rate was 62.2% (range: nurses 28.5%; physicians 69%) among the 990 healthcare workers approached/targeted. The participant groups included nurses (n=47), general practitioners (n=102), consultants (n=99), senior or chief residents (n=52), junior residents (n=236) and interns or senior house officers (n=80). The clinical post-registration experience of most respondents fell within 0 to 5 years or 6 to 11 years across most groups of healthcare workers except for consultants, most of whom had experience of ≥ 24 years. Most respondents practiced at 1 of the 2 major hospitals (UHWI or KPH) in the Kingston and St. Andrew area which includes the nation’s capital city, and several respondents were based at other smaller hospitals. While most respondents worked in the public healthcare system, several healthcare workers also work in private healthcare settings. A notable exception was the group of general practitioners, most of whom had practices based in outpatient and other settings. Unsurprisingly, the highest levels of postgraduate training and sepsis training were found in consultants, senior or chief residents and junior residents.

### Assessment of Attitudes Towards Sepsis Management

An overwhelming majority of respondents agree that more training on sepsis is needed (93.7%) and that sepsis bundles should be implemented at their respective hospital or practice (93.2%) (Table 1). Most nurses (97.9%), interns/Senior House Officers (SHO) (96.3%), Senior/Chief Residents (88.5%), Residents (86.0%) and General Practitioners (GPs) (85.3%) indicated that more sepsis training is needed. While a majority of Consultants (72.7%) also thought more sepsis training was needed, this was significantly lower than other respondent groups (p=0.014). Similarly, while the majority of Consultants (77.8%) and General Practitioners (81.4%) also thought that sepsis bundles should be implemented at their hospitals or practice, these were significantly lower than majorities in other respondent groups, which ranged from 89.0 to 97.9%, p=0.003.

**Table 1:** Summary of Attitudes to Sepsis

Questions	Groups	Clinical Positions N (%)							
		All (n=616)	Nurse (n=47)	GP (n=102)	Consultant (n=99)	Senior/ Chief Resident (n=52)	Resident (n=236)	Intern/ SHO (n=80)	p-value
Q15. Need More	Yes	577(93.7)	46(97.9)*	87(85.3)	**72(72.7)**	46(88.5)	203(86)	77(96.3)*	
Sepsis Training?	No	37(6.0)	0(0)	5(4.9)	10(10.1)	4(7.7)	12(5.1)	1(1.3)	0.014

Q16. Extent HCW	Strong Agree	148(24)	15(31.9)	23(22.5)	20(20.2)	8(15.4)	61(25.8)	13(16.3)	0.131
Can Identify Sepsis	Somewhat Agree	311(50.5)	15(31.9)	46(45.1)	41(41.4)	32(61.5)	113(47.9)	43(53.8)	0.064
Risk?	Neutral	106(17.2)	14(29.8)	18(17.6)	14(14.1)	6(11.5)	33(14)	18(22.5)	0.095
	Somewhat Disagree	29(4.7)	2(4.3)	3(2.9)	7(7.1)	2(3.8)	8(3.4)	4(5)	0.509
	Strong Disagree	4(0.6)	**0(0)**	2(2.0)	**0(0)**	2(3.8)*	**0(0)**	**0(0)**	0.016

Q17. Implement	Yes	574(93.2)	46(97.9)*	**83(81.4)**	**77(77.8)**	49(94.2)	210(89.0)	78(97.5)*	
Sepsis Bundle? Training	No	20(3.2)	0(0)	9(8.8)	5(5.1)	1(1.9)	5(2.1)	0(0)	0.003

The clinical group(s) indicating least agreement with the indicated question is (are) highlighted in bold and statistical comparison is made with that group(s) as the baseline reference. (*p<0.05, **p<0.01 compared to reference group).

Interestingly, despite the variation in the attitudes towards the need for more sepsis training, there was very good agreement across respondent groups with respect to the extent to which healthcare workers can identify patients at risk for sepsis. Most respondents across participant groups agreed somewhat that Health Care Workers (HCWs) can identify these at-risk patients, while most others either strongly agreed or were neutral. Only a few Senior/Chief Residents (3.8%) and GPs (2.0%) strongly disagreed with this assertion.

### Assessment of Knowledge and Practice of Sepsis Management

**Fig. 1:**
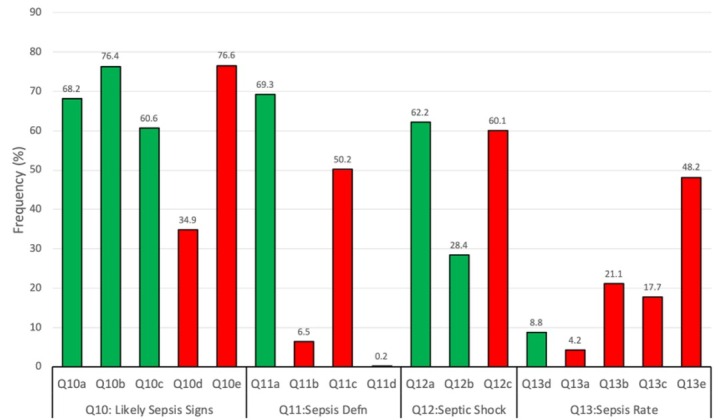
Frequency of respondents indicating correct and incorrect knowledge responses (Q 10-13). Correct responses (green) are indicated first followed by incorect responses (red) for each question.

**Fig. 2:**
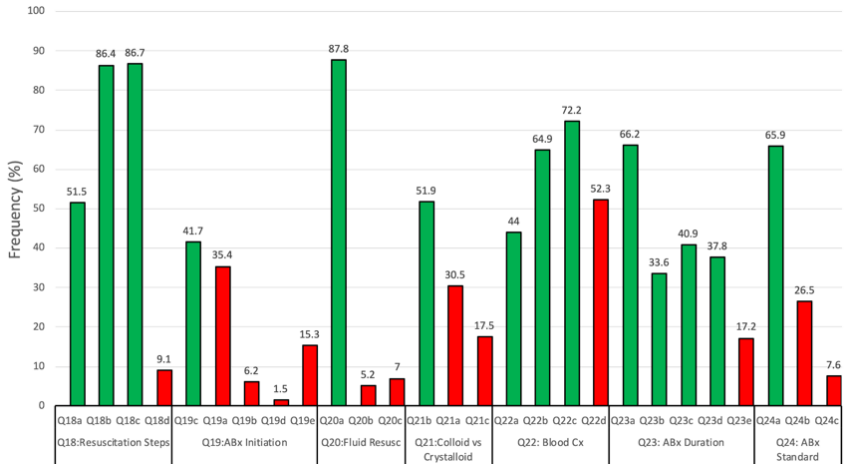
Frequency of respondents indicating correct and incorrect practice responses (Q 18-24). Correct responses (green) are indicated first followed by incorect responses (red) for each question.

Among all respondents, correct answers to both knowledge and practice questions were more frequent than incorrect answers (Figures 1 and 2). With respect to knowledge assessment, respondents were able to correctly identify low systolic BP, altered mental status and tachypnoea as likely signs of sepsis by qSOFA in 60.6 to 76.4% of cases, while only 34.9% of respondents incorrectly indicated that a low PaCO2 was a likely sign of sepsis. However, many respondents (76.6%) incorrectly indicated that an abnormal White Blood Cell (WBC) as a criterion for qSOFA. Similarly, most respondents (69.3%) correctly defined sepsis as a dysregulated host infection response, while incorrect responses ‘blood poisoning’ and ‘allergic reaction’ were indicated by 6.5% and 0.2% of respondents, respectively. However, just over half of respondents (50.2%) incorrectly indicated that the presence of bacteremia was an adequate definition for sepsis. The need for vasopressors (62.2%) was correctly indicated by most respondents as a feature of septic shock but far fewer respondents (28.4%) correctly identified high serum lactate levels as such. In contrast, most respondents (60.1%) incorrectly believed that sepsis associated with cardiovascular dysfunction was an indication of septic shock. Quite surprisingly, only a few respondents (8.8%) were able to identify the correct worldwide annual sepsis mortality rate of 20 to 50%.

With respect to the practice assessment, most respondents were able to correctly identify measuring lactate levels (51.5%), blood cultures before antibiotics (86.4%) and broad-spectrum antibiotic therapy (86.7%) as essential steps in sepsis management in the first 3 hours of presentation. Only a few participants (9.1%) incorrectly indicated that blood transfusion to correct a hypotensive state was such a measure. While a plurality of respondents (41.7%) were correct that 1 hour was the appropriate interval within which antibiotic therapy could be successfully administered after a presumptive sepsis diagnosis, only slightly fewer participants (35.4%) indicated the incorrect interval of 20 minutes. Much fewer respondents indicated that either 45 minutes (6.2%) or 35 hours (1.5%) was the correct interval but 15.3% of respondents indicated that they were unaware of the correct response. An overwhelming majority (87.8%) and just over half (51.9%) of respondents correctly indicated that fluid resuscitation was necessary before Intensive Care Units (ICUs) admission and that colloid fluids were NOT preferable to crystalloid fluid respectively. Fewer study participants (5.2% and 30.5%, respectively) indicated the opposite response, while 7.0% and 17.5% of respondents respectively indicated a lack of awareness of the correct practice on either question. Most respondents correctly selected hypothermia (64.9%) and neutropenia (72.2%) as indications for obtaining a blood culture while fewer respondents (44.0%) correctly identified the presence of chills as such an indication. Close to half (52.3%) of participants incorrectly identified a neutrophil right shift as an indication for blood culture. Most respondents (66.2%) correctly identified an undrainable infection focus as an indication for longer antibiotic therapy duration, however much fewer correctly identified *S.aureus* bacteremia (33.6%), neutropenia (40.9%) or some fungal infection (37.8%) in the same regard. Almost a fifth (17.2%) of respondents indicated that they were not aware of the correct practice. The typical antibiotic therapy duration of 7 to 10 days was correctly identified by most respondents (65.9%), while 26.5% incorrectly answered the opposite and 7.6% indicated that they did not know the correct duration.

### Assessment of Knowledge and Practice Quantitative Scores

Among all study participants, mean scores (95% CI) for correct knowledge (max 7), incorrect knowledge (max 7), correct practice (max 14) and incorrect practice (max 7) were 3.7 (3.6-3.9), 3.2 (3.1-3.3), 8.3 (8.1-8.5) and 2.3 (2.2-2.4), respectively. There was no significant difference in correct knowledge, correct practice, and incorrect practice scores between male and female respondents. However, male respondents scored slightly lower in the incorrect knowledge score (3.0[2.8-3.2]) compared to female respondents (3.3[3.2-3.4], p<0.01).

Across clinical groups, nurses scored the lowest in correct knowledge (2.6[2.1-3.1]) and correct practice (6.2[5.4-7.0]) scores and consultants, interns/SHO, residents and senior/chief residents all had significantly higher correct knowledge (p<0.01) and correct practice (p<0.01) scores. GPs also had significantly higher practice scores compared to nurses (p<0.05) but slightly lower than other groups. Conversely, senior/chief residents and junior residents were the lowest scoring groups in incorrect knowledge (2.6[2.2-3.0]) / 2.9[2.7-3.1], respectively) and practice scores (1.8(1.42.1] / 2.0[1.8-2.2], respectively. Consultants (p<0.01), GPs (p<0.01) and nurses (p<0.01) all had significantly higher incorrect knowledge and practice scores.

### Years post registration experience

With respect to classification according to years of experience post-registration there was no significant difference in correct knowledge scores (range 3.6 to 4.1). However, respondents that had 6-11 years post registration experience scored the highest in correct practice scores (9.2[8.8-9.5]), which was significantly higher than those with 0-5 yrs experience (p<0.01), and ≥ 24yrs experience (p<0.01).

Conversely, respondents with 6-11yrs experience had the lowest incorrect knowledge (2.9[2.7-3.1]) and incorrect practice (1.8[1.6-2.0]) and participants with 18-23 yrs experience (p<0.05) and ≥ 24yrs experience (p<0.01) had higher incorrect knowledge scores. All other experience groups had significantly higher incorrect practice scores.

### Post graduate training and sepsis training

Both post-graduate training and sepsis training were indicators of higher correct knowledge scores (4.0[3.84.2] / 3.9[3.7-4.1], respectively) and correct practice scores (8.8[8.5-9.1] / 8.8[8.5-9.1], respectively) compared to those without this training (p<0.01/p<0.05). Conversely, respondents with post-graduate training (2.2[2.0-2.4]) and sepsis training (2.1[2.0-2.3]) had lower incorrect practice scores compared to those without this training (p<0.01), however there was no statistical difference with respect to incorrect knowledge scores among those with or without either of these types of training.

### Specialty

Respondents who specialized in Anaesthesia/ Critical Care Medicine (4.8[4.1-5.6]) and Emergency medicine (5.0[4.4-5.5]) had the highest correct knowledge scores (p<0.05) compared to those in Paediatrics (3.1[2.53.6]) and Public Health (3.1[1.9-4.2]), who scored the lowest. These 2 groups of respondents, Anaesthesia/ Critical Care Medicine (2.2[1.2-3.3]) and Emergency medicine (2.2[1.5-2.9]) also had the lowest incorrect knowledge scores, while those in Obstetrics & Gynaecology (3.7[3.3-4.1], p<0.05), Public Health (3.7[3.24.3], p<0.05), Psychiatry (3.9[3.2-4.5], p<0.05) and Family medicine (3.9[3.1-4.8], p<0.01) had the highest incorrect knowledge scores.

Respondents specializing in Internal Medicine (10.1[9.3-10.8)] scored highest in correct practice (p<0.01), compared to those in Psychiatry (6.6[4.88.5]), Family Medicine (7.5[5.8-9.2]), and other specialties (7.5[5.9-9.0]). Respondents specializing in Psychiatry had the highest incorrect practice scores (3.9(3.0-4.7]) compared to those in Anaesthesia/Critical Care Medicine, Emergency Medicine, Haematology/Pathology, Internal medicine, Paediatrics and Surgery who all had significantly lower incorrect practice scores (p<0.01).

### Independent Predictive Variables of Knowledge and Practice Quantitative Scores

With respect to independent predictors of high and low values for each of the 4 quantitative scores after elimination of confounding factors: the clinical specialty, the clinical position, and the years of post-registration experience of the respondent were the most influential predictive factors (Table 2).

**Table 2:** Multivariate Linear Regression Models Outlining Independent Predictors for Correct and Incorrect Knowledge and Practice Scores

Scores	Independent Predictors	LS Mean Score	B coeff (95%CI)	p-value	(R^2^ model adjusted R^2^)
Correct Knowledge	Anaesth/Critical Med	5.1	1.1(0.1/2.1)	0.027	0.20 (0.15)
	Emergency Med	4.8	0.8(0.1/1.6)	0.045	
	Other Specialty	3.2	-0.8(-0.1/-1.6)	0.047	
	Psychiatry	3.0	-1.1(-0.1/-2.1)	0.040	
	Public Health	2.9	-1.1(-0.1/-2.2)	0.034	
	Family Med	2.8	-1.2(-0.1/-2.2)	0.031	
	Paediatrics	2.8	-1.2(-0.6/-1.8)	<0.001	
	Nurse	2.2	-1.7(-0.8/-2.7)	0.001	

Incorrect Knowledge	General Practitioner	3.8	1.2(0.5/1.9)	0.001	0.20 (0.16)
	O&G	3.8	0.8(0.3/1.3)	0.001	
	Paediatrics	3.7	0.7(0.2/1.2)	0.004	
	Consultant	3.5	0.8(0.4/1.3)	0.001	

Correct Practice	Internal Medicine	9.5	1.5(0.6/2.4)	0.001	0.21 (0.16)
	General Practitioner	7.2	-1.9(-0.6/-3.2)	0.006	
	Exp 0-5yrs	7.2	-1.4(-0.7/-2.2)	<0.001	
	Nurse	5.7	-3.3(-1.8/-4.9)	<0.001	
	Psychiatry	5.7	-2.2(-0.7/-3.8)	0.005	

Incorrect Practice	Psych	3.9	1.5(0.6/2.3)	0.001	0.25 (0.20)
	Nurse	3.8	1.6(0.7/2.4)	<0.001	
	Exp>24yrs	3.2	1.2(0.6/1.8)	<0.001	
	No Sepsis Training	2.8	0.3(0.1/0.6)	0.034	
	Exp 0-5yrs	2.7	0.6(0.2/1.1)	0.004	
	Exp 12-17yrs	2.7	0.7(0.2/1.2)	0.005	

LS Mean Score (Least Square Mean Score): The mean score after controlling for confounding variables. B coeff (95%CI) – Beta coefficient (95% confidence interval): a measure of the degree of change in the outcome variable for every unit of change in the predictor variable. R^2^ is a measure of goodness of fit of the regression model.

Anaesthesia/ Critical Care Medicine and Emergency Medicine specialties were independent predictors of high correct knowledge scores while unnamed specialties, Psychiatry, Public Health, Family Medicine, Paediatrics specialties and Nurse clinical position were independent predictors of low correct knowledge scores. This multivariate linear regression model accounted for 20% of the variability noted in the incorrect knowledge score in the cohort.

The clinical positions of GP and Consultant and O&G and Paediatrics specialties were all independent predictors of high incorrect knowledge scores. This multivariate linear regression model accounted for 20% of the variability noted in the incorrect knowledge score in the cohort.

Internal Medicine specialty was the only independent predictor of a high correct practice score. GP and Nurse clinical positions, Psychiatry, and those with 0-5 years post-registration experience were independent predictive factors for low correct practice scores. This multivariate linear regression model accounted for 21% of the variability noted in the correct practice score in the cohort.

Psychiatry specialty, nurse clinical position, no sepsis training and post-registration experience of 0-5yrs, 12-17yrs and ≥ 24yrs were all independent predictors of high incorrect practice scores. This multivariate linear regression model accounted for 25% of the variability noted in the incorrect practice score in the cohort.

## Discusssion

This study is the first in the region, evaluating the knowledge, practice, and attitudes towards the management of sepsis by healthcare workers in Jamaica. The recent update to the Surviving Sepsis Guidelines for Management of Sepsis and Septic Shock (SSC Guidelines) provides best practice principles for the management of sepsis patients in a hospital setting [17]. The assessment of physician’s knowledge related to sepsis management in other countries has highlighted overall poor knowledge of SSC guidelines, with an improvement in both physician knowledge and practice following performance improvement programmes for sepsis being put into effect [18]. The implementation of SSC bundles (a core set of recommendations) in hospitals has been associated with decreased mortality in several studies [19]. Lower mortality was observed in hospitals with higher compliance, rates decreasing 0.7% for every 3 months that a hospital participated in the SSC [20].

Previous surveys about the knowledge of nurses and doctors about sepsis guidelines indicated that it was poor or inadequate [11,14,21, 22, 23]. Our results demonstrate that healthcare workers’ knowledge of sepsis management principles is most complete among the Anaesthesia/Critical Care and Emergency Medicine specialties. This is perhaps not surprising given the frequency with which these groups of healthcare workers encounter critically ill patients with sepsis. Therefore, these clinicians are much more likely to be aware of recommended SSC guidelines, which has been noted in previous studies in other countries [14,24]. Similarly, the Internal Medicine specialty had the highest sepsis practice scores, again not a surprise given that sepsis management outside the ICU setting most often falls under the purview of these physicians. Indeed, our multivariate linear regression model highlighted Anaesthesia/Critical Care and Emergency Medicine as independent predictors of high sepsis knowledge scores and Internal Medicine as an independent predictor of high sepsis practice scores. Of note, physician specialties with less frequent direct encounters with critically ill patients and non-physician categories of healthcare workers had significantly lower knowledge and practice scores. All these data argue strongly for the benefit of SSC bundle implementation since continuous exposure to sepsis management principles and guidelines seems most likely to translate into improved knowledge and practice by healthcare workers.

Other key factors that predict good sepsis knowledge and practice are the extent of training as well as specific training geared towards sepsis management and the seniority of positions.

While persons 6 - 11 years post-registration had the highest sepsis practice scores, there was no significant difference in knowledge scores across groups of varying time post-registration.

The fact that sepsis practice scores decrease after 11 years post-registration and that there was no difference in knowledge scores across the groups highlights the overall need for training in all groups of respondents with a focus on retraining to maintain correct sepsis management principles and practice even in physicians with specialty training.

Both post-graduate and sepsis training were indicators of higher correct knowledge and correct practice scores in our study, which were in contrast to several previous studies [11,18]. A study population of 91 internal resident doctors showed no association between years of experience and knowledge [18] compared to the findings in our study. Possible reasons include application of guidelines of the Surviving Sepsis Campaign (SSC), improvements in training or other unknown time-dependent factors. In our study population only 24.4% heard of SSC. The results of this study showed that over 90% agree that more training on sepsis is needed and implementation of sepsis bundles at their institution is necessary despite their own understanding of the principles and practice of sepsis management.

Most of the persons (69.3%) could correctly define sepsis as a dysregulated host infection response, which is comparative to a smaller study in 2003 which found that 54% of training grade doctors could recognize the definition of the stage of sepsis [13]. On the contrary, Poeze et al. reported much lower figures of only 22% among Intensivists and 5% among Medical Specialists [11]. The low numbers in that study were obtained using an opened ended question to evaluate the definition of sepsis unlike our study where we utilized a closed ended question for that item. They also found that no more than 17% of the physicians agreed on any one definition of sepsis and that at least six different definitions were mentioned by at least 1 in 10 of the physicians [11]. This difference in our study could have resulted from improvement in knowledge due to more active teaching programmes. There is, however, a lack of clarity and consistency about sepsis definitions among doctors [13]. The definition of sepsis and septic shock is widely agreed to be somewhat confusing and often subject to change with increasing knowledge of the pathophysiology of the dysfunction [21,25]. Indeed, as these last SSC guidelines were being developed, new definitions for sepsis and septic shock were published, highlighting the role of life-threatening organ dysfunction and a dysregulated immune response in the process [16]. Interestingly, most of our respondents correctly identified the need for vasopressors as a key component of septic shock, while also incorrectly indicating that sepsis associated with cardiovascular dysfunction was an indication of septic shock. This very much highlights the difficulty several healthcare workers have in identifying the underlying concepts of sepsis pathophysiology. This misinformation could lead to confusion in the management of sepsis at presentation and as a corollary can increase morbidity and mortality of these patients [4, 5, 6, 7]. This is important as when sepsis presents, it requires similar immediate intervention as myocardial infarction [26].

Only a small number of respondents (less than 10%) knew the correct worldwide annual sepsis mortality rate, which was significantly less compared to >40% reported in Puerto Rico [12]. However, our assessment indicated correct responses with respect to sepsis bundle guidelines for the initial 3 hours in sepsis management in most respondents. The measurement of lactate levels, blood cultures being taken before antibiotics, and the use of broad-spectrum antibiotic therapy were correctly identified as key components of sepsis management by most respondents, which indicated a good level of practice for the initial management of sepsis among most healthcare workers interviewed. The practice to have adequate fluid resuscitation before ICU admission was also known by most respondents. The indications for obtaining blood cultures were recognized in most respondents, which shows that this tool of diagnosing sepsis is recognized. However, the indications for longer duration of antibiotic therapy were less well known by respondents, which could potentially lead to incorrect or inappropriate antibiotic use and as such the development of antibiotic resistance in dangerous pathogens.

There are some limitations of this study. The total number of nurses was disproportionately lower than physicians which was due to the low response rate among this group of workers. Therefore, our results are not generalizable to nurses in Jamaica. Also, we used a convenience sampling method to recruit participants which has its inherent selection bias. However, we sought to ensure that all categories of physicians from interns to consultants were represented. The strength of this study is that it includes a wide range of participants from the major hospitals and outpatient settings across the island of Jamaica. We used the 2016 International Guidelines for Management of Sepsis and Septic Shock which were current during the study period. Therefore, our study reflected the knowledge and practice of healthcare workers based on the then current international guidelines as a standard of practice. Those guidelines were subsequently updated in 2021 to call for the utilization of different sepsis screening tools and to clarify the need for immediate antimicrobial therapy. Interestingly, those changes did not significantly affect the correct responses to our questions.

## Conclusion

In a survey, it was observed physicians who were Anaesthetists or worked in Critical Care/Emergency medicine had the greatest knowledge of sepsis. Evidence is shown that more sepsis education is required for healthcare workers in Jamaica. The management of sepsis may benefit from implementation of SSC bundles in Jamaica.
